# Supplementary material for: Spatiotemporal refinement of signal flow through association cortex during learning
Source: Nat Commun. 2020 Apr 8;11:1744. doi: 10.1038/s41467-020-15534-z (PMC7142160; doi:10.1038/s41467-020-15534-z)
Supplement: Supplementary file 1 — Supplementary Information [file 41467_2020_15534_MOESM1_ESM.pdf]

Supplementary Information:

Title:

Spatiotemporal refinement of signal flow through association cortex during learning

Gilad et al.

Supplementary Figures

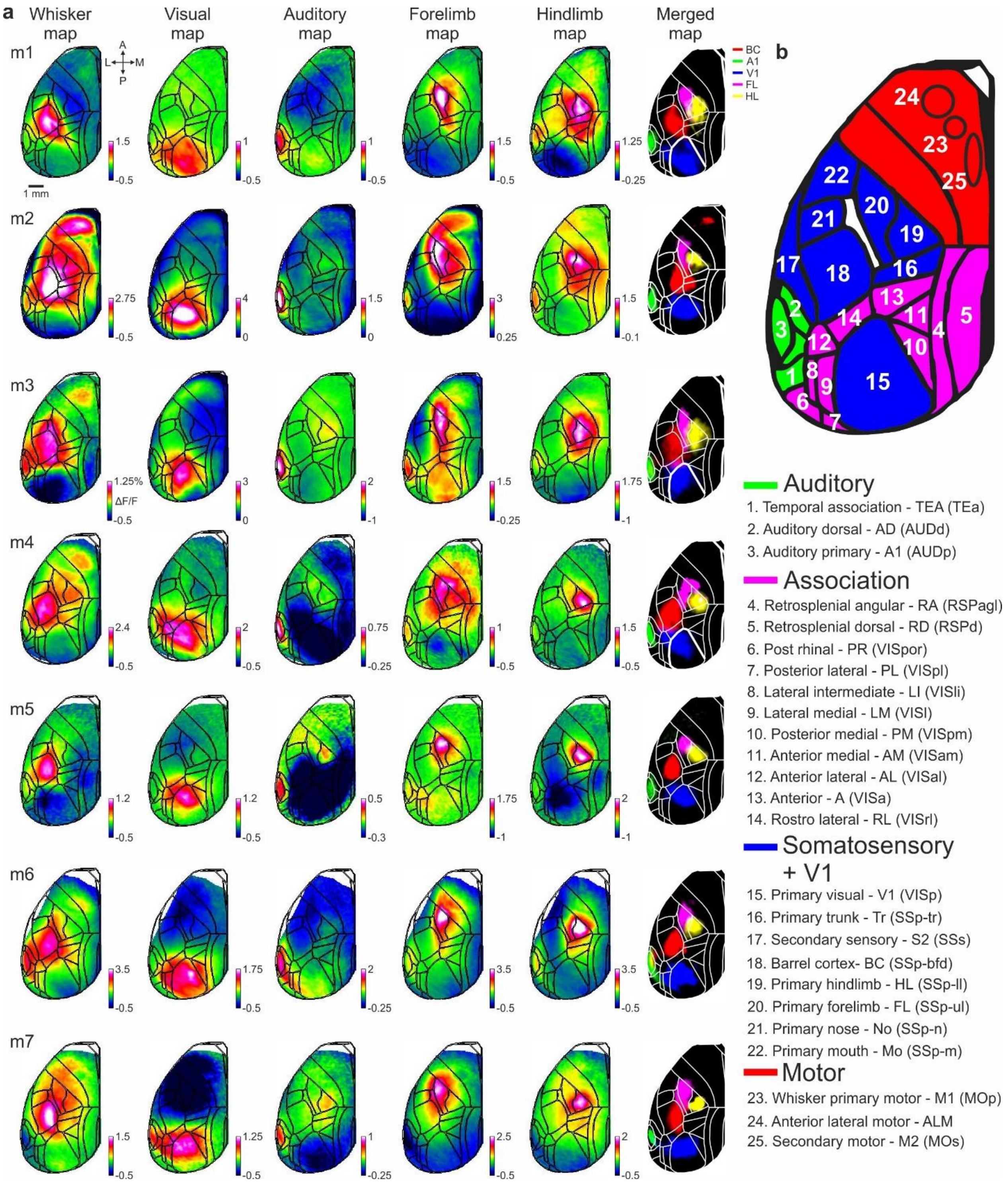

Supplementary Figure 1 │ Functional mapping and area definitions. a, Each mouse underwent a mapping session under anesthesia including stimulation for five sensory modalities: whisker, visual, auditory, forelimb and hindlimb (Methods). Mean activation maps are shown for each stimulus type for all 7 mice. Color denotes normalized fluorescence (ΔF/F). Furthermore, maps were registered onto a top 2D view of the mouse

atlas (black lines; ©2004 Allen Institute for Brain Science. Allen Mouse Brain Atlas. Available from: http://mouse.brain-map.org/). Overlays of the 5 maps are shown on the right. **b,** Full names and abbreviations of all the 25 areas used in this study. For comparison, the abbreviations used by the Allen Institute are included in brackets. Areas were divided into auditory (green), association (pink), somatosensory + V1 (blue) and motor (red) cortices.

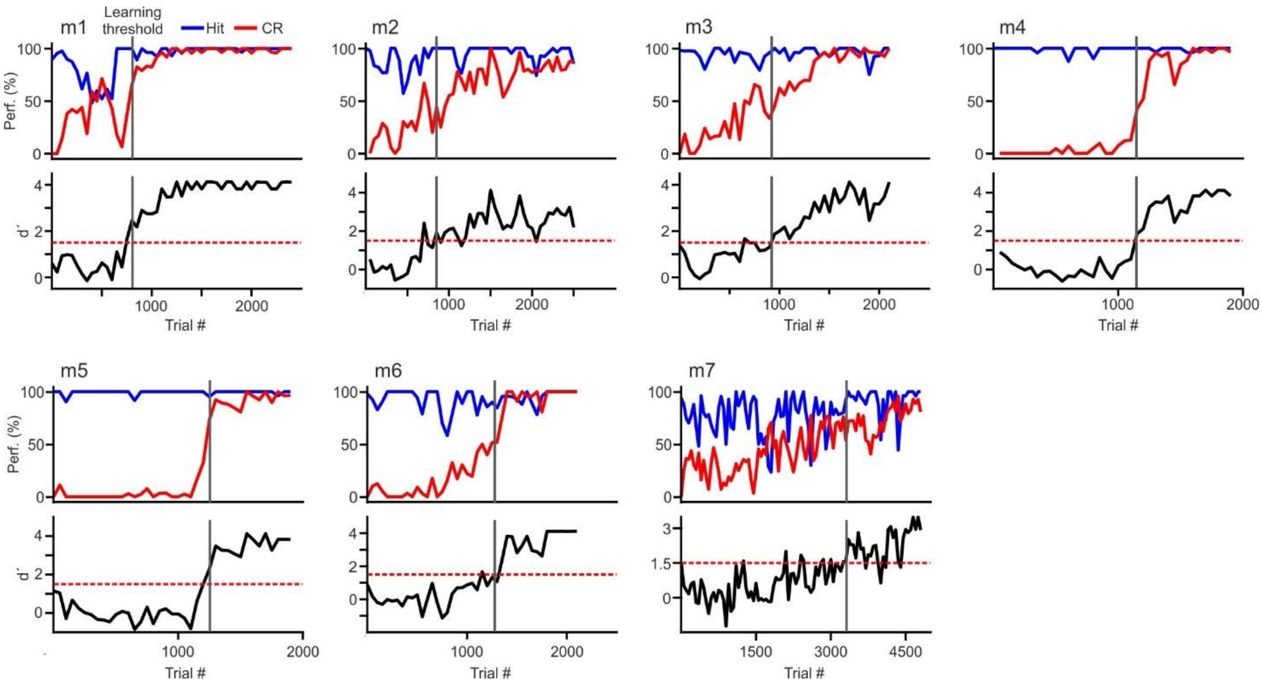

Supplementary Figure 2 │ Behavioral performance and learning curves. Behavioral performance for all seven mice, plotting the time course of Hit and CR rates in percent (top) and d' (bottom) as a function of trial number. The learning threshold when mice reached d' = 1.5 is indicated by vertical gray lines.

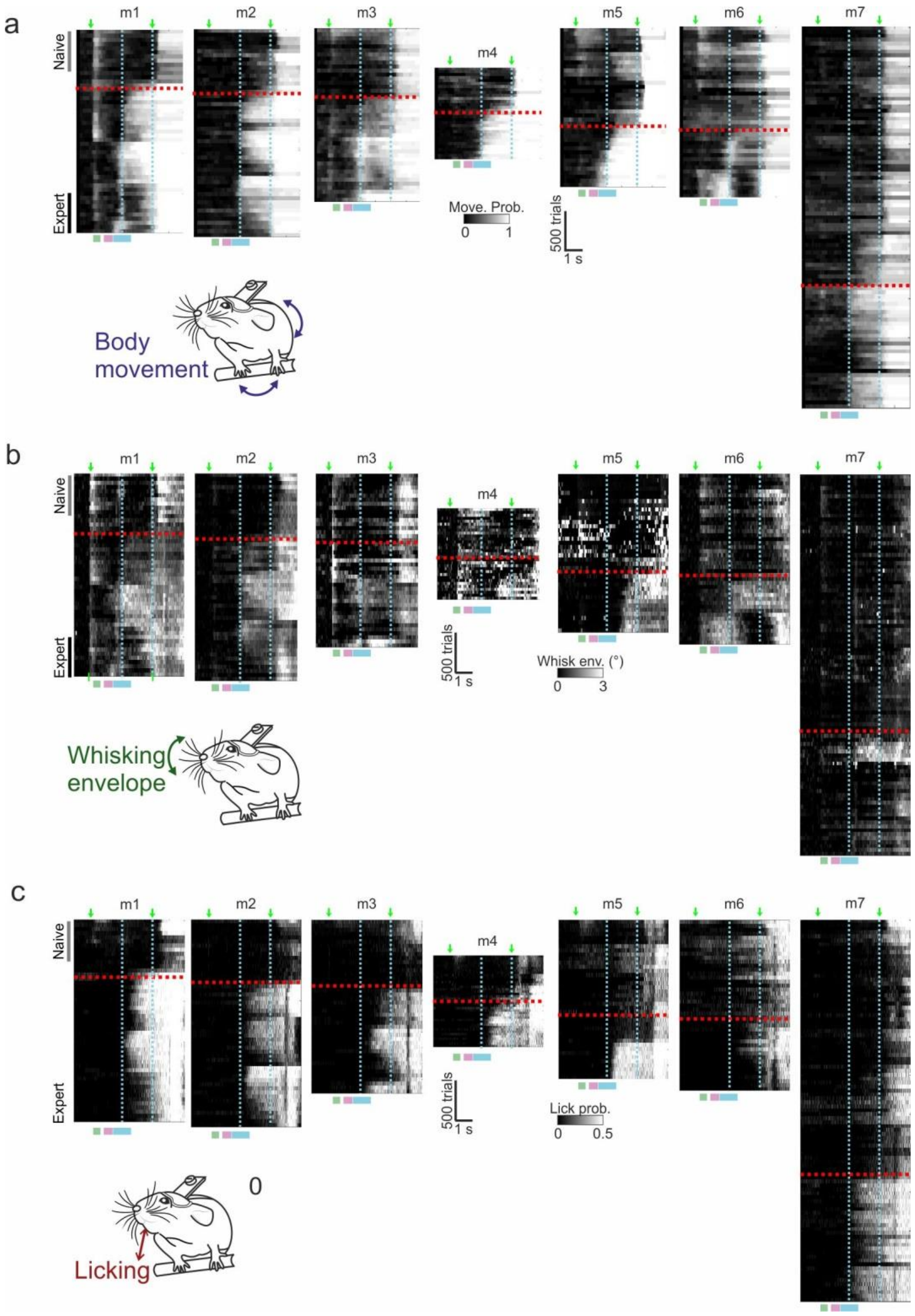

Supplementary Figure 3 │ Behavioral changes during learning. a, Body movements quantified as movement probability throughout the trial time across learning for all 7mice (50-trial bins; similar to Fig. 2a). Dashed red line indicates learning threshold. Heat maps are vertically aligned to this learning threshold. Dashed cyan lines indicate the texture-in period. Green arrows mark stimulus and response cues. **b,** Equivalent heat maps as in a but for the trial-related whisking envelope dynamics. **c,** Equivalent heat maps as in a but for the trial-related licking probability.

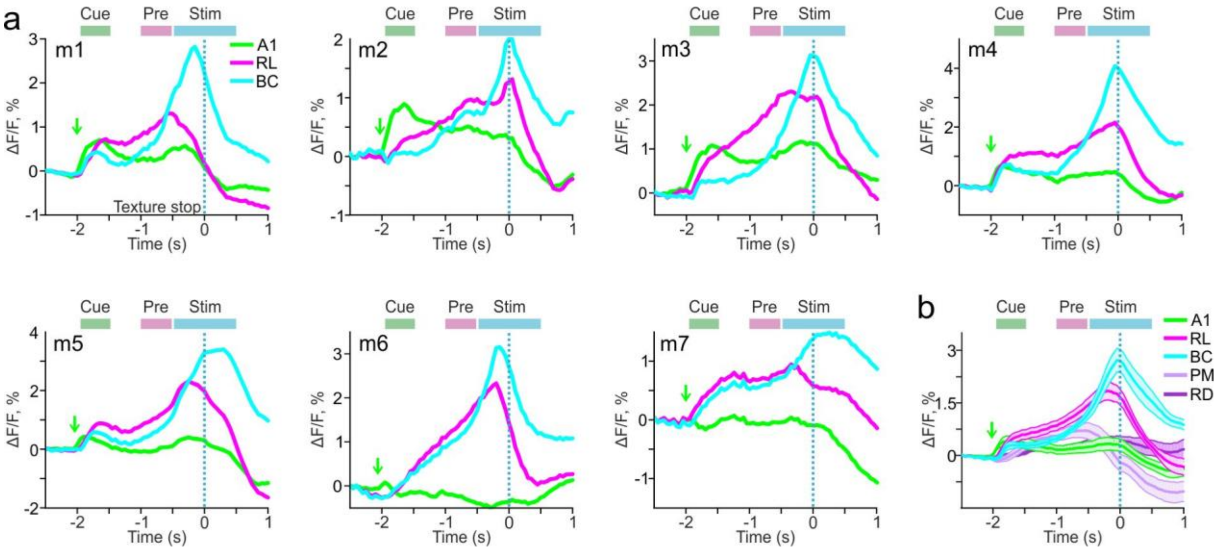

Supplementary Figure 4 │ Expert trial-related responses in different areas for all 7 expert mice. **a**, Responses in A1, RL and BC for each expert mouse separately. Green arrow marks stimulus cue. Similar to Figure 3b. **b**, Responses in A1, RL, PM, RD and BC averaged across all mice. Error bars are s.e.m. across mice.

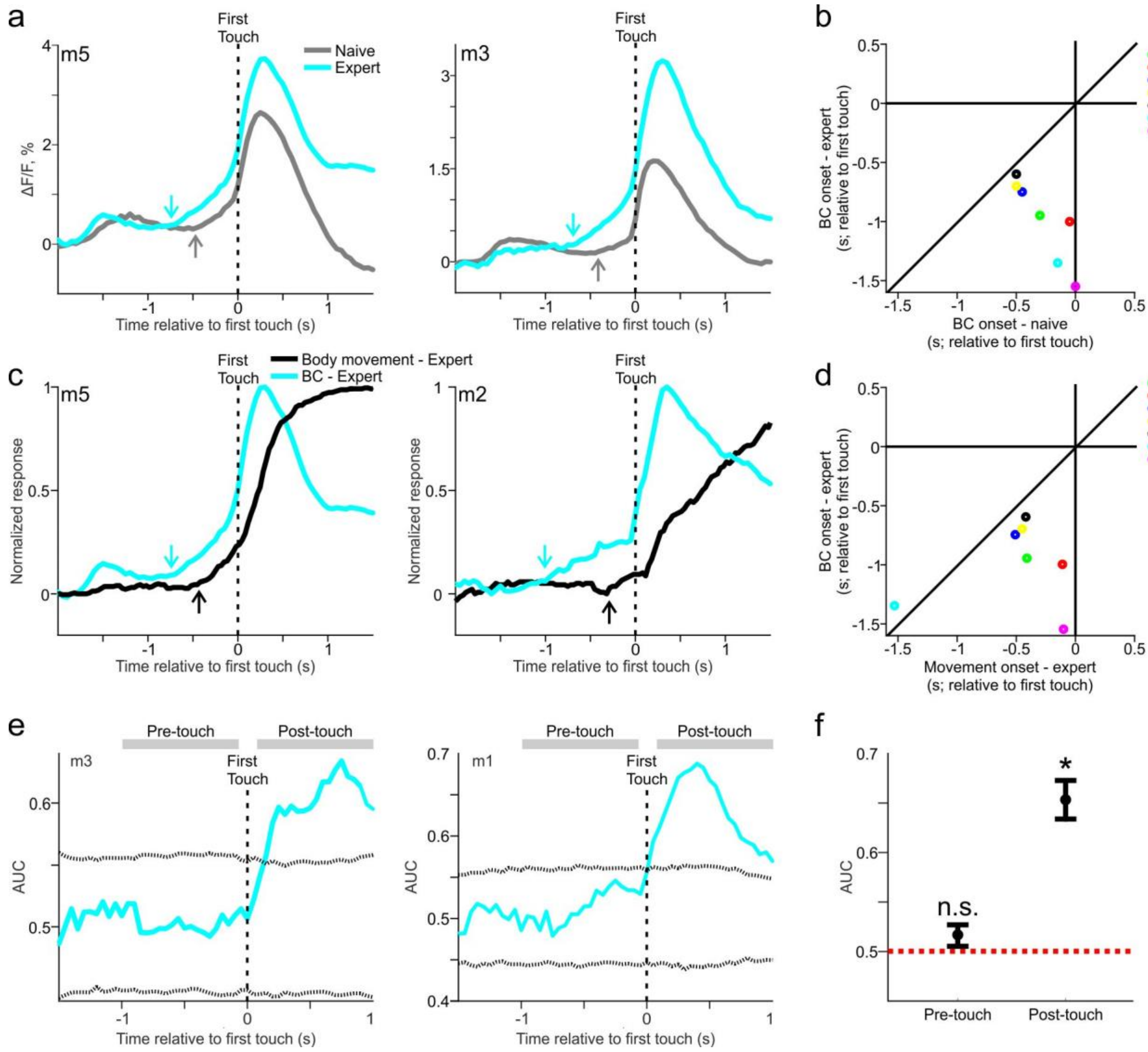

Supplementary Figure 5 │ Anticipatory activity in BC is enhanced during learning. **a,** BC response aligned to the first touch of the whiskers on the incoming texture (time 0 marks texture touch) for naïve (gray) and expert (cyan) in two example mice. Onset of responses (i.e. onset of initial rise) are marked with arrows. **b,** Response onsets in BC for naïve vs expert in all 7 mice. **c,** BC response (cyan) plotted against body movement vector (black) in two example expert mice. Both curves are normalized between 0 and 1. BC response and movement onsets are marked in arrows. **d,** BC response versus the movement onsets for all expert mice. **e,** ROC-AUC values for go vs. no-go trials for BC responses aligned on the first touch (time 0). Examples from two mice (for m1 the plot is equivalent to Figure 6b where BC responses are not aligned to first touch). Dashed gray lines indicate mean ± 2 s.d. of shuffled data. **f,** AUC values averaged across all mice during pre and post touch (gray bars in e). Error bars are s.e.m. across mice. $^*p < 0.05$; n.s. – not significant; Wilcoxon signed-rank test.

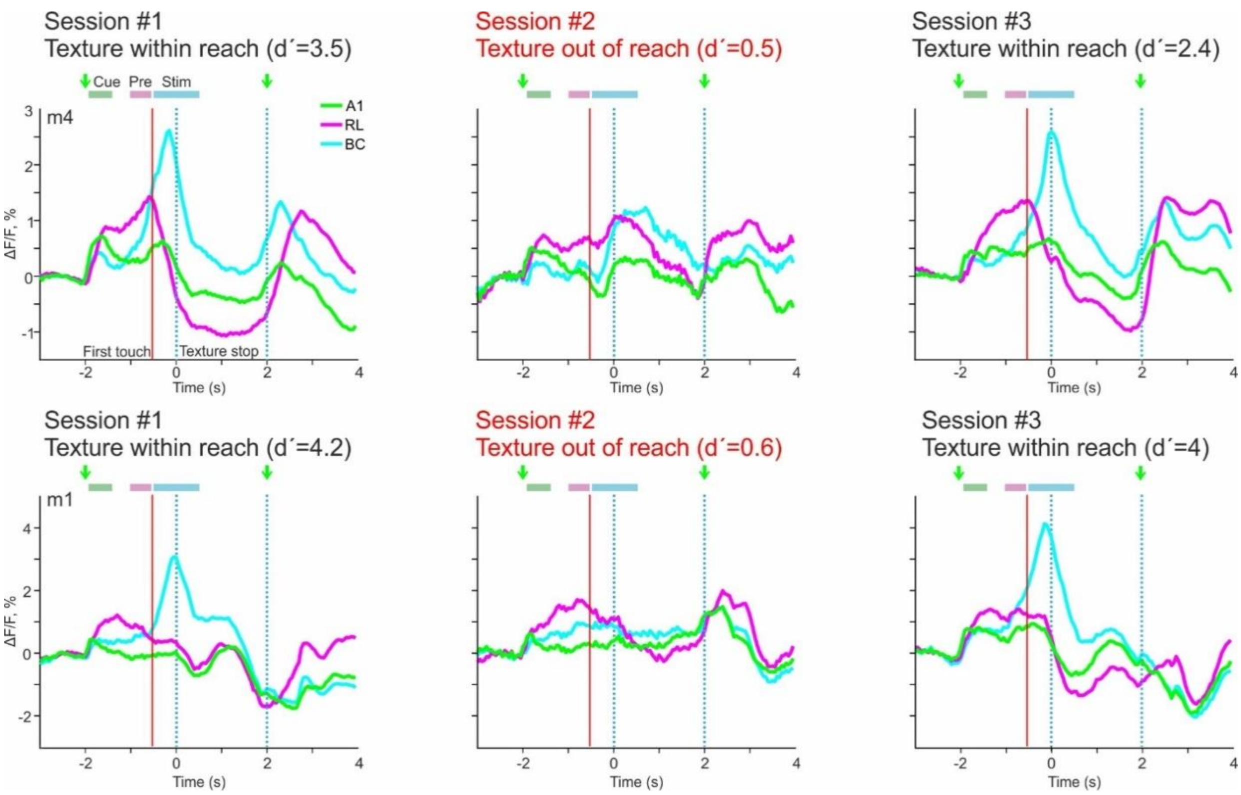

Supplementary Figure 6 │Cortical activation responses in expert mice with the texture out of reach. In two mice that reached expert level we performed wide-field calcium imaging when the texture was placed just out of reach of the whiskers. Here we plot the cortical signals in go trials for A1 (green traces), RL (magenta), and BC (cyan), comparing a session with texture out-of-reach (session #2) with the previous and the subsequent session, when the texture was within normal reach (sessions #1 and #3). Note in particular the strong activation response in RL during the pre-period, which persisted even when the texture was out-of-reach. In contrast, the texture-touch related activation observed in BC was abolished (as well as the subsequent suppression of RL and A1 during touch). The activations at the start of texture removal were also reduced although interestingly a response to the stimulus-cue only remained in RL in session #2.

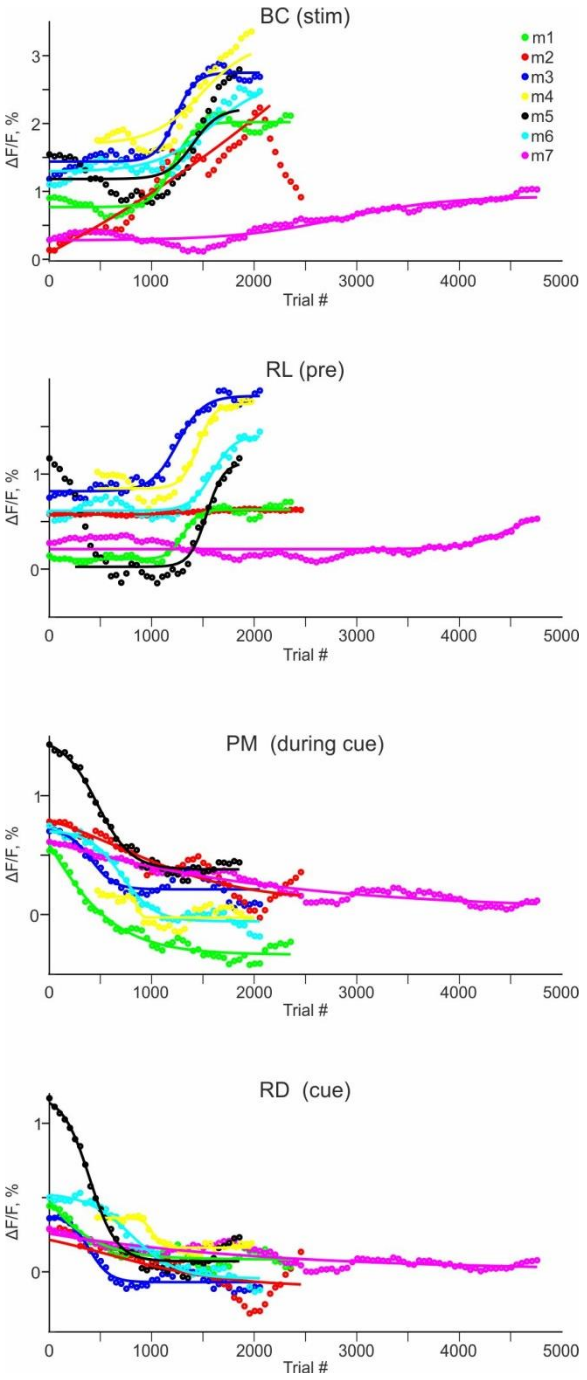

Supplementary Figure 7 │ Examples of non-normalized learning-related activity changes. Learning-related changes in mean DF/F signals for four selected areas for each mouse (different colors). From top to bottom: BC during stim-period, RL during pre-period, PM during cue-period and RD during cue-period. Data were fitted with sigmoidal curves.

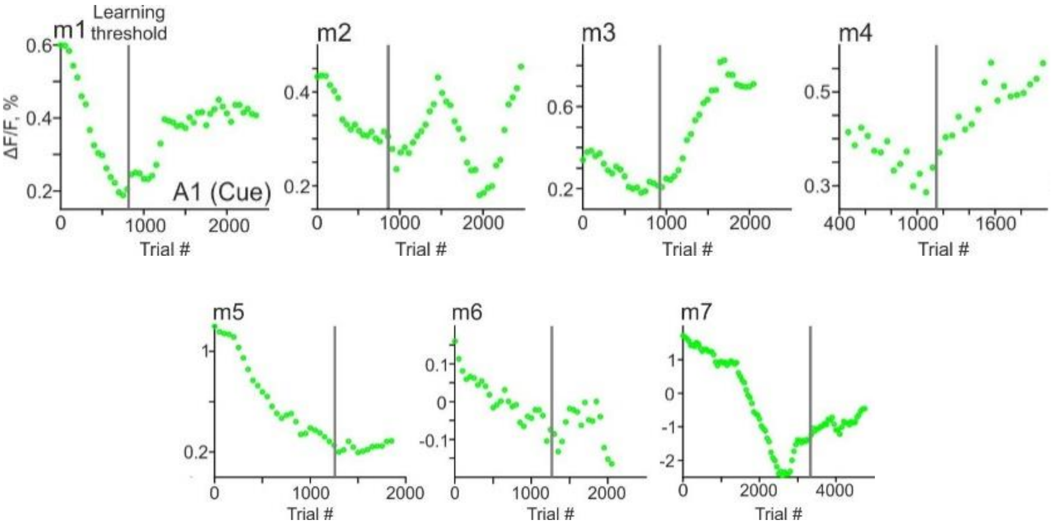

Supplementary Figure 8 │ Variability of learning-related changes in A1. Time course of learning-related changes in ΔF/F signals in A1 during the cue-period for all 7 mice. Learning thresholds are indicated with vertical gray lines. Note that suppression consistently occurs before the learning threshold while enhancement thereafter.

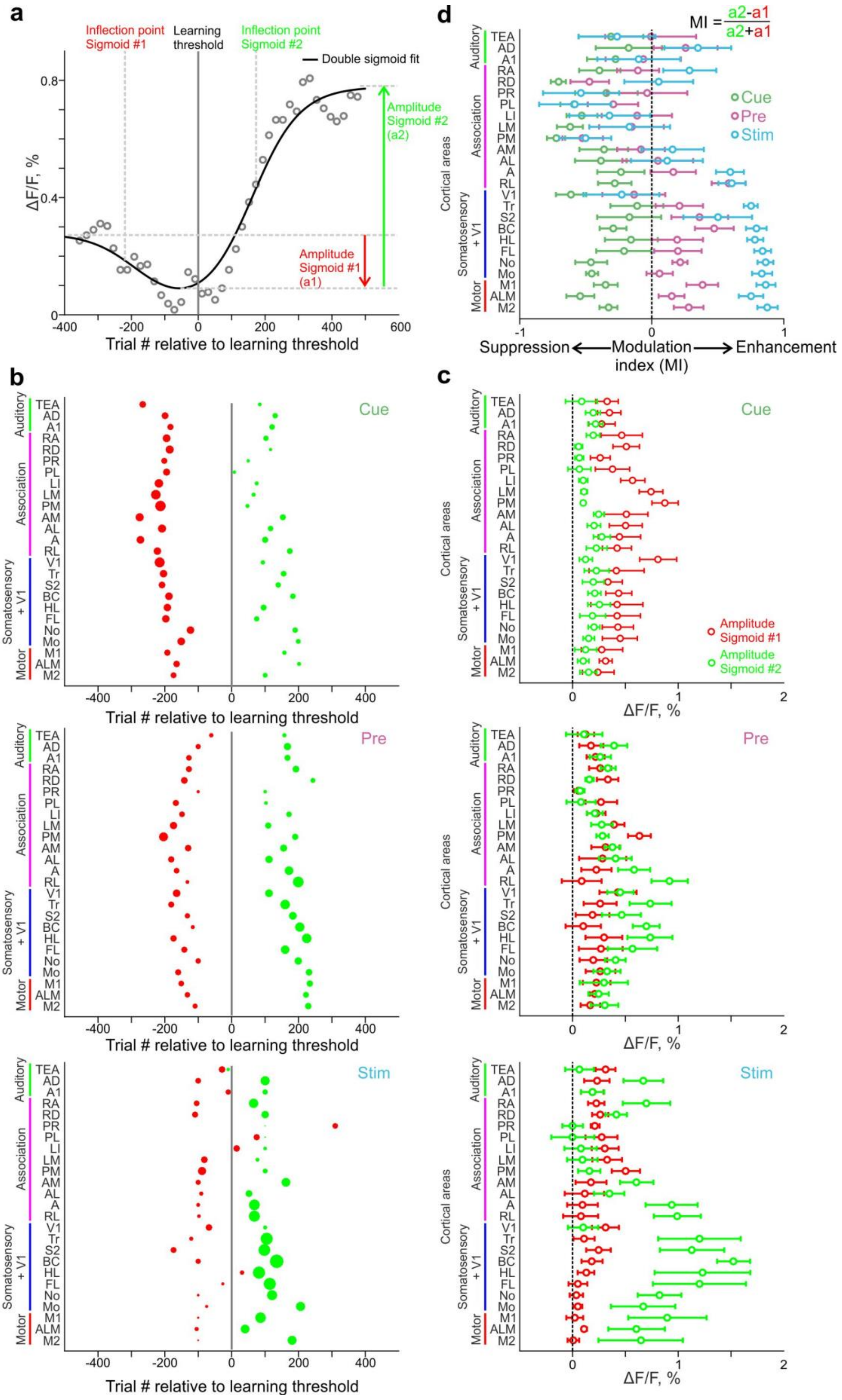
a
Inflection point Sigmoid #1
Learning threshold
Inflection point Sigmoid #2
Double sigmoid fit
Amplitude Sigmoid #2 (a2)
Amplitude Sigmoid #1 (a1)
ΔF/F, %
Trial # relative to learning threshold
b
Cue
Pre
Stim
Cortical areas
Auditory
Association
Somatosensory + V1
Motor
TEA
AD
A1
RA
RD
PR
PL
LI
LM
PM
AM
AL
A
RL
V1
Tr
S2
BC
HL
FL
No
Mo
M1
ALM
M2
Trial # relative to learning threshold
c
Amplitude Sigmoid #1
Amplitude Sigmoid #2
ΔF/F, %
d
MI = (a2-a1)/(a2+a1)
Cue
Pre
Stim
Suppression
Modulation index (MI)
Enhancement

Supplementary Figure 9 | Quantification of suppression and enhancement in the pre-learning and learning phases for all areas and trial periods. a. An example learning curve from a given area (gray points) along with a double-sigmoid fit (black curve) comprised of a decreasing and an increasing sigmoid function (red and green, respectively). Four parameters are depicted: the amplitudes and the inflection points of the sigmoids. **b.** Grand average of amplitudes and latencies for the decreasing (red) and increasing (green) sigmoid for each area during the cue-, pre-, and stim-periods. Inflection points are arranged relative to the learning thresholds with the circle diameter proportional to the sigmoid amplitude. **c.** Amplitudes of each sigmoid for all areas in the cue-, pre- and stim-periods. Error bars are s.e.m across mice. In some cases, it is also shown that a good prediction of the learning threshold is the minimal $\Delta F/F$ values at the turning point between the sigmoids. **d.** Modulation index to quantify enhancement/suppression ratio for each area and each trial period. Positive values indicate predominant enhancement whereas negative values relate to predominant suppression. Error bars as in c.

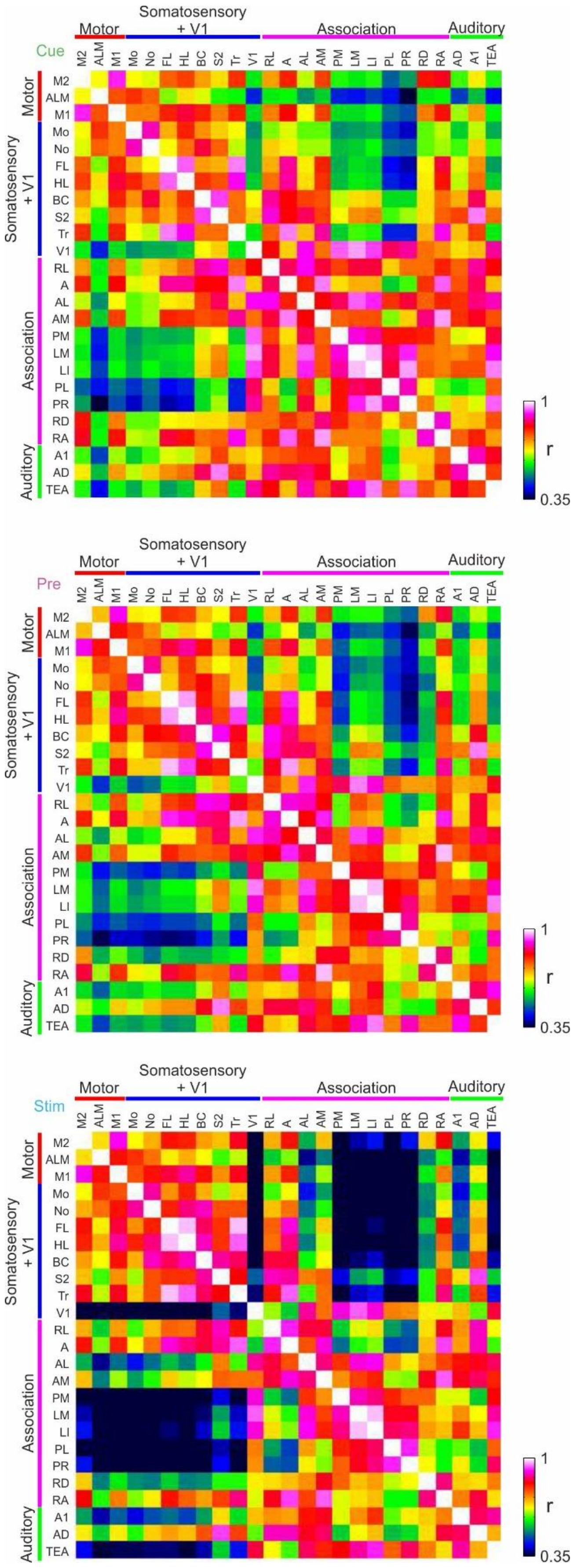
Cue
Pre
Stim
Motor
Somatosensory + V1
Association
Auditory
M2 ALM M1 Mo No FL HL BC S2 Tr V1 RL A AL AM PM LM LI PL PR RD RA AD A1 TEA
M2 ALM M1 Mo No FL HL BC S2 Tr V1 RL A AL AM PM LM LI PL PR RD RA A1 AD TEA
r
1
0.35

Supplementary Figure 10 │ Correlation matrices between learning curves of different areas. Full correlation matrix for all 25 areas, showing the pair-wise correlation coefficient between ΔF/F activity curves of two areas during cue (top), pre (middle) and stim (bottom) periods.

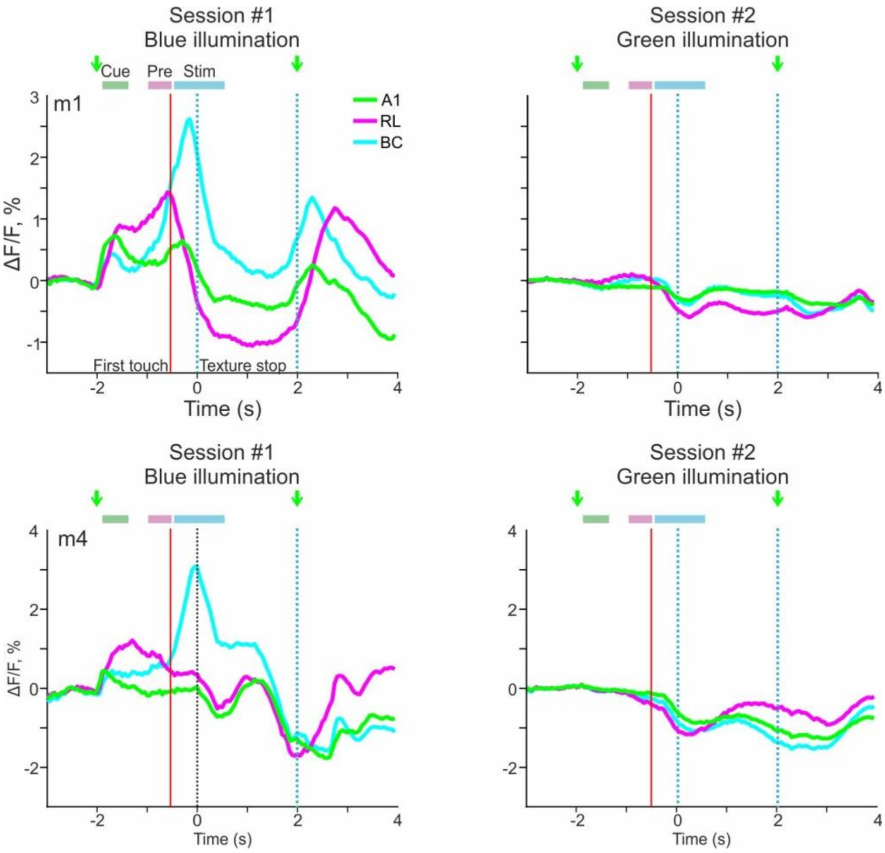

Supplementary Figure 11 │ Controls for non-calcium related optical signals. In two mice that reached expert level, we controlled for non-calcium related signal by exciting the wide-field preparation with green light, which is more related to hemodynamic signals. Displayed are the responses in BC, RL and A1 with green light (session #2) compared to the normal excitation with blue light (session #1). There are no major fluorescence changes in the green control, especially during cue and pre periods.
